# Supplementary figures and images for: Mahalanobis distances for ecological niche modelling and outlier detection: implications of sample size, error, and bias for selecting and parameterising a multivariate location and scatter method
Source: PeerJ. 2021 May 11;9:e11436. doi: 10.7717/peerj.11436 (PMC8121071; doi:10.7717/peerj.11436)

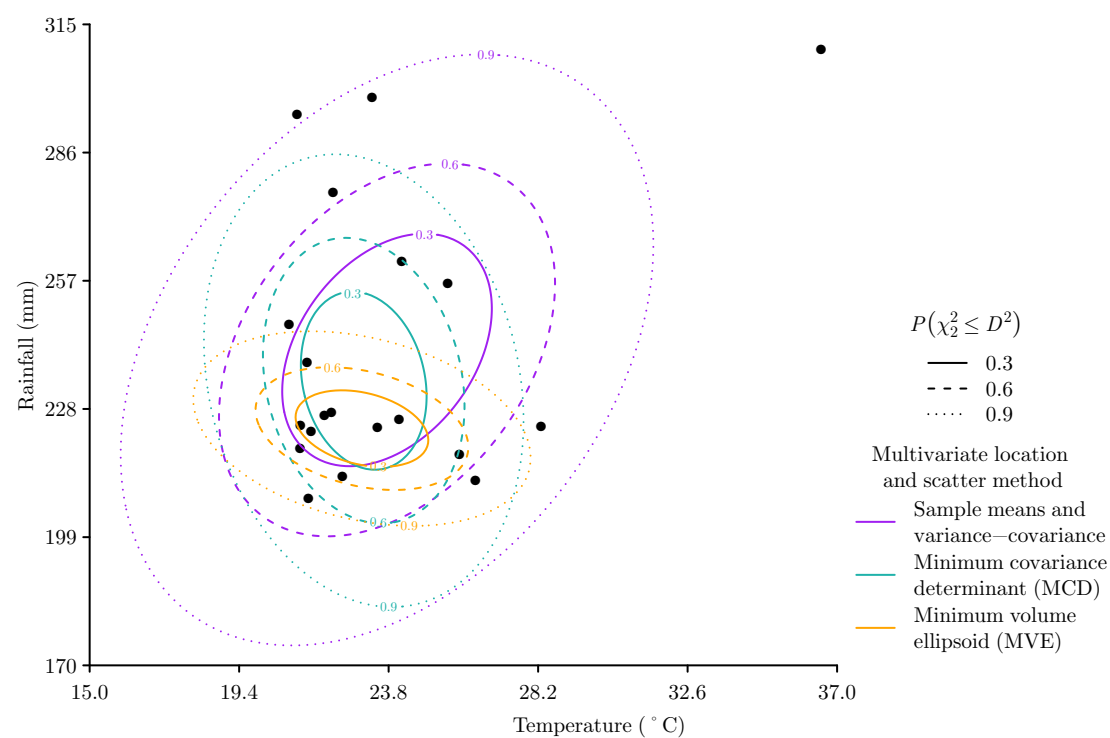

Supplement: Supplemental Information 1 [file peerj-09-11436-s001.zip › 3-figures/figure1/figure1.pdf]

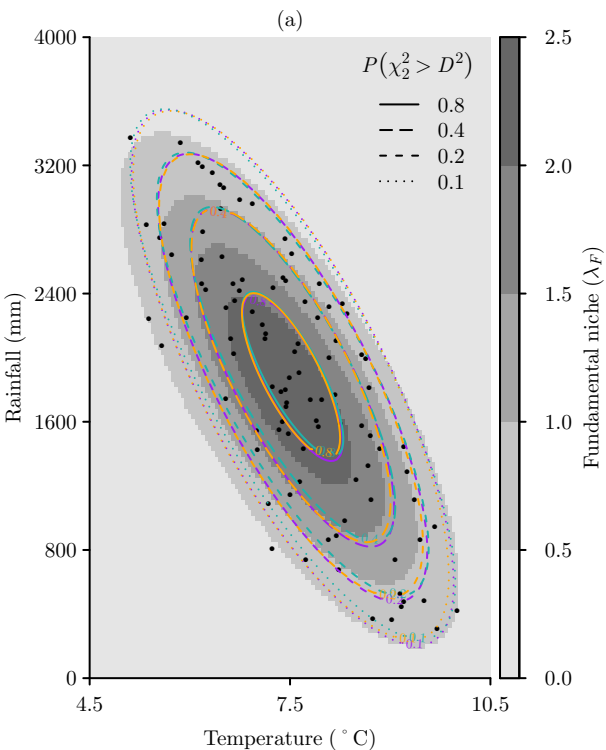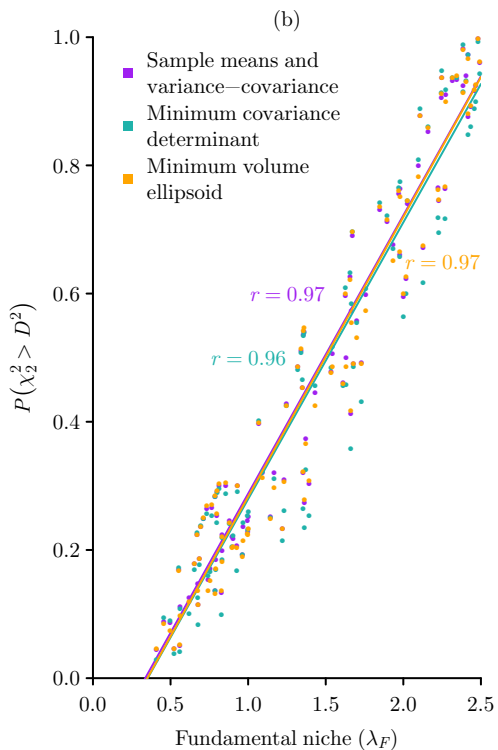

Supplement: Supplemental Information 1 [file peerj-09-11436-s001.zip › 3-figures/figure2/figure2.pdf]

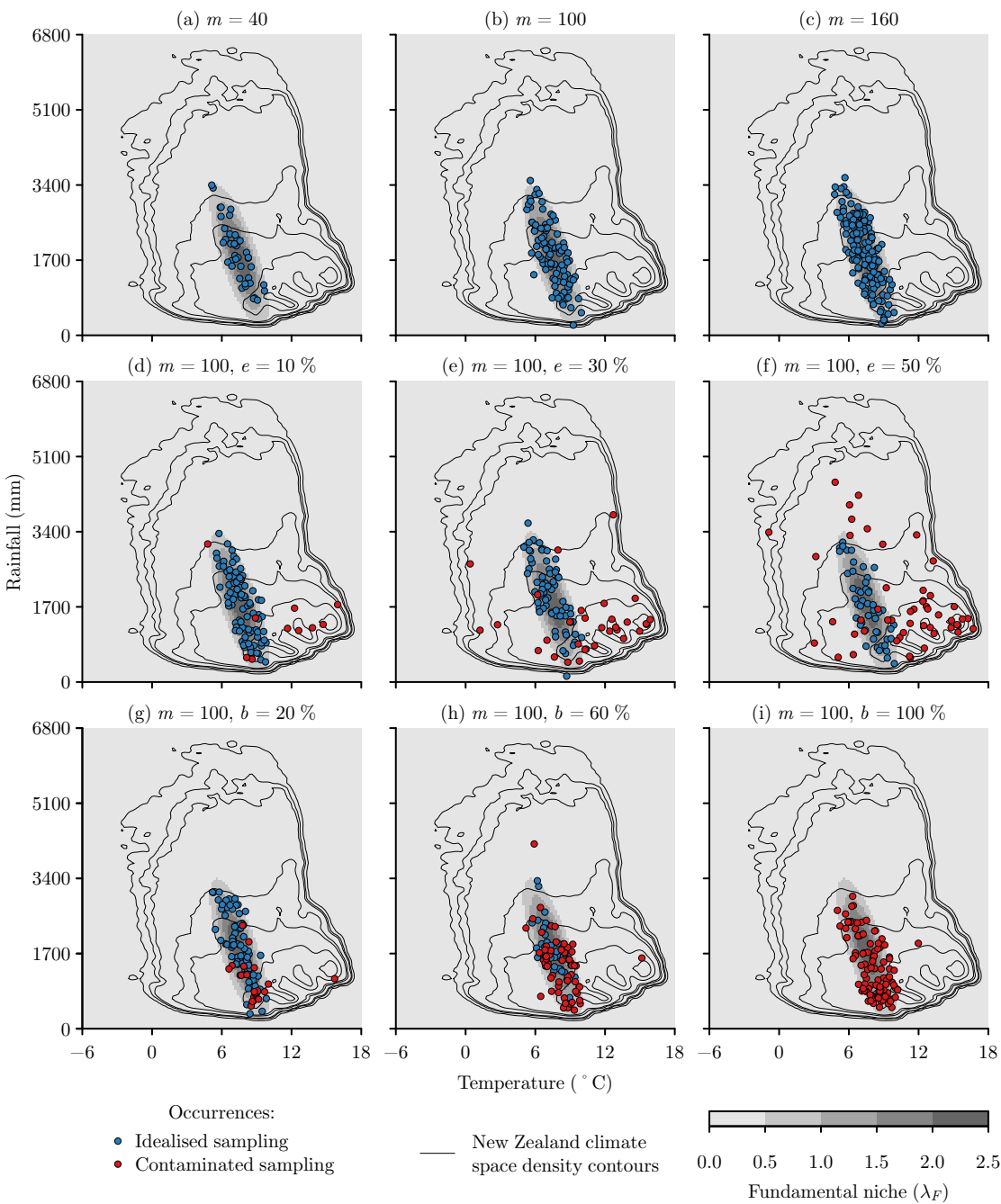

Supplement: Supplemental Information 1 [file peerj-09-11436-s001.zip › 3-figures/figure3/figure3.pdf]

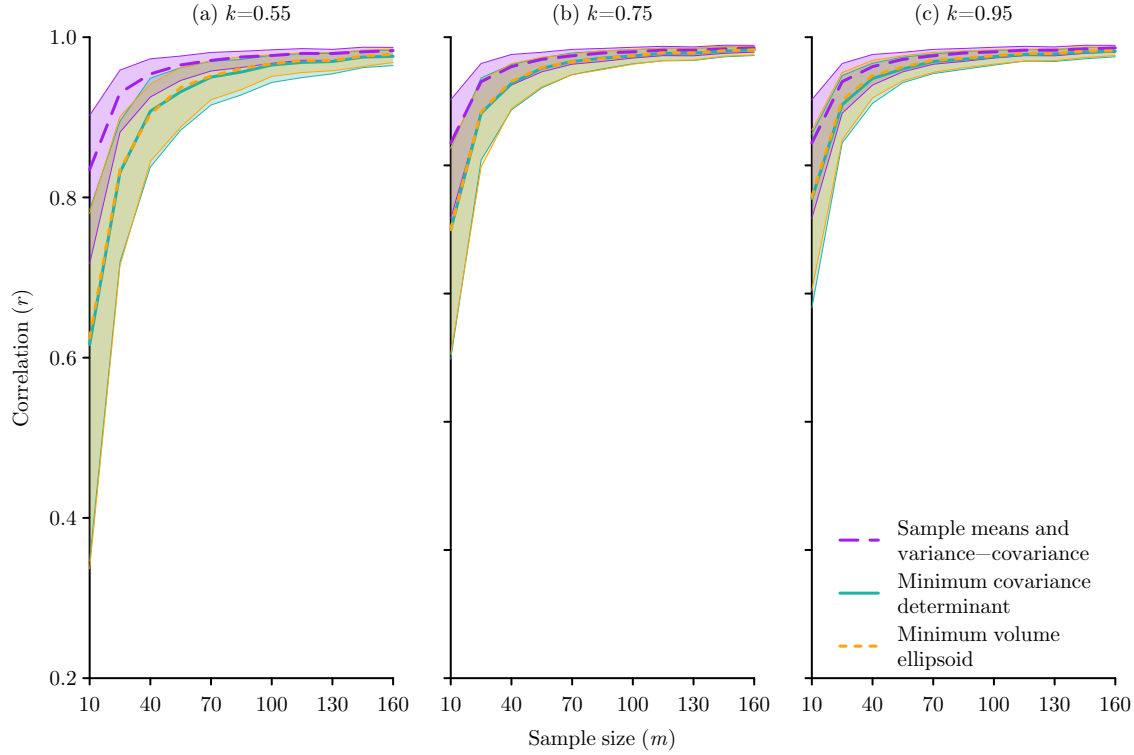

Supplement: Supplemental Information 1 [file peerj-09-11436-s001.zip › 3-figures/figure4/figure4.pdf]

(a)  $k=0.55$ 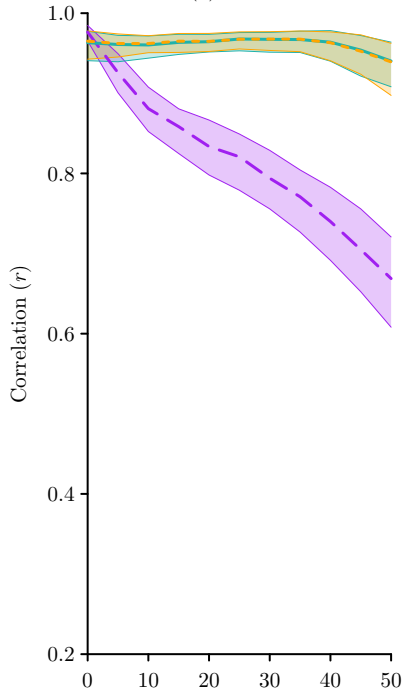(b)  $k=0.75$ 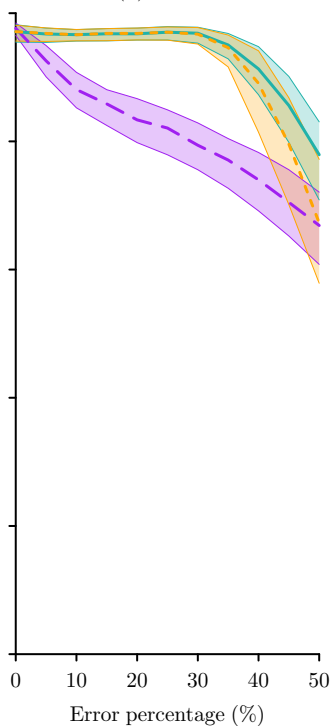(c)  $k=0.95$ 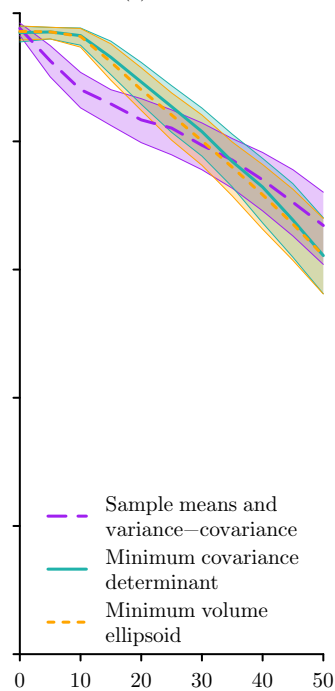

Supplement: Supplemental Information 1 [file peerj-09-11436-s001.zip › 3-figures/figure5/figure5.pdf]

(a)  $k=0.55$ 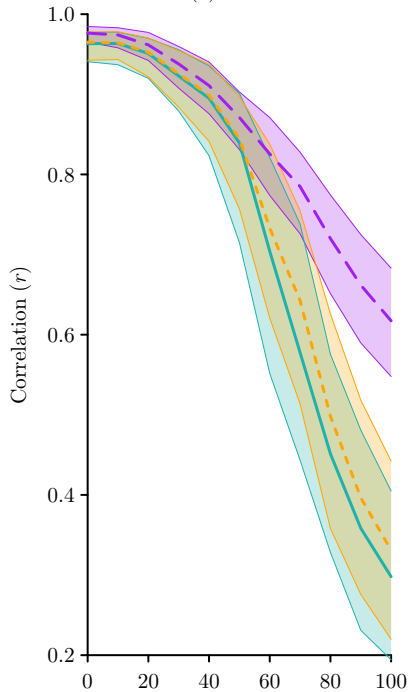(b)  $k=0.75$ 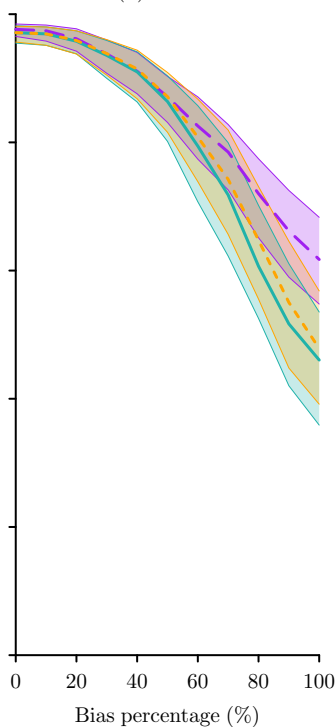(c)  $k=0.95$ 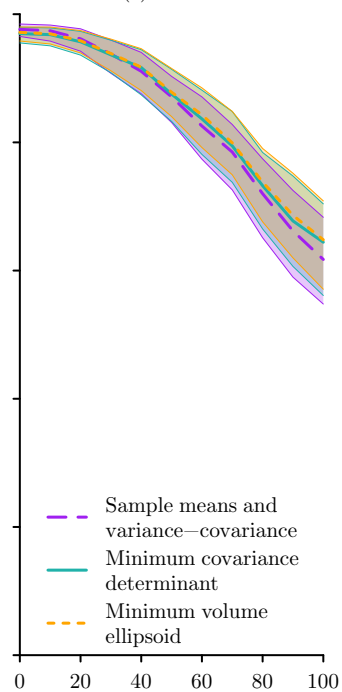

Supplement: Supplemental Information 1 [file peerj-09-11436-s001.zip › 3-figures/figure6/figure6.pdf]
